# Propensity Score Methods in Health Technology Assessment: Principles, Extended Applications, and Recent Advances

**DOI:** 10.3389/fphar.2019.00973

**Published:** 2019-09-18

**Authors:** M Sanni Ali, Daniel Prieto-Alhambra, Luciane Cruz Lopes, Dandara Ramos, Nivea Bispo, Maria Y. Ichihara, Julia M. Pescarini, Elizabeth Williamson, Rosemeire L. Fiaccone, Mauricio L. Barreto, Liam Smeeth

**Affiliations:** ^1^Faculty of Epidemiology and Population Health, London School of Hygiene and Tropical Medicine, London, United Kingdom; ^2^Nuffield Department of Orthopaedics, Rheumatology and Musculoskeletal Sciences (NDORMS), Center for Statistics in Medicine (CSM), University of Oxford, Oxford, United Kingdom; ^3^Centre for Data and Knowledge Integration for Health (CIDACS), Instituto Gonçalo Muniz, Fundação Osvaldo Cruz, Salvador, Brazil; ^4^GREMPAL Research Group (Idiap Jordi Gol) and Musculoskeletal Research Unit (Fundació IMIM-Parc Salut Mar), Universitat Autònoma de Barcelona, Barcelona, Spain; ^5^University of Sorocaba–UNISO, Sorocaba, São Paulo, Brazil; ^6^Institute of Public Health, Federal University of Bahia (UFBA), Salvador, Brazil; ^7^Department of Statistics, Federal University of Bahia (UFBA), Salvador, Brazil

**Keywords:** bias, confounding, effectiveness, health technology assessment, propensity score, safety, secondary data, observational study

## Abstract

Randomized clinical trials (RCT) are accepted as the gold-standard approaches to measure effects of intervention or treatment on outcomes. They are also the designs of choice for health technology assessment (HTA). Randomization ensures comparability, in both measured and unmeasured pretreatment characteristics, of individuals assigned to treatment and control or comparator. However, even adequately powered RCTs are not always feasible for several reasons such as cost, time, practical and ethical constraints, and limited generalizability. RCTs rely on data collected on selected, homogeneous population under highly controlled conditions; hence, they provide evidence on efficacy of interventions rather than on effectiveness. Alternatively, observational studies can provide evidence on the relative effectiveness or safety of a health technology compared to one or more alternatives when provided under the setting of routine health care practice. In observational studies, however, treatment assignment is a non-random process based on an individual’s baseline characteristics; hence, treatment groups may not be comparable in their pretreatment characteristics. As a result, direct comparison of outcomes between treatment groups might lead to biased estimate of the treatment effect. Propensity score approaches have been used to achieve balance or comparability of treatment groups in terms of their measured pretreatment covariates thereby controlling for confounding bias in estimating treatment effects. Despite the popularity of propensity scores methods and recent important methodological advances, misunderstandings on their applications and limitations are all too common. In this article, we present a review of the propensity scores methods, extended applications, recent advances, and their strengths and limitations.

## Introduction

Randomized clinical trials (RCTs) are generally accepted as the gold-standard approaches for measuring the “causal” effects of treatments on outcomes (Sibbald and Roland, 1998; Concato et al., 2000) and the design of choice for health technology assessment (HTA). In causal inference terminology using Rubin’s potential outcomes framework (Rubin, 2005), the effect of a certain treatment (Z = 1) versus a control or comparator (Z = 0) on an outcome (Y) involves comparison of potential outcomes under treatment (Y_1_)) and an alternative treatment (Y_0_)). In RCT, with sufficient numbers of participants and adequate concealment of allocation, randomization ensures that individuals assigned to treatment and control or comparator groups are comparable in all pretreatment characteristics, both measured and unmeasured (Sibbald and Roland, 1998). The only difference is that one group received the treatment (Z = 1) and the other received no treatment or the alternative treatment (Z = 0); hence, any difference in outcomes between the two groups can be attributable to the effect of the treatment. In other words, the “causal” effect of treatment in the study population (the average treatment effect, ATE) on outcomes can be estimated by a direct comparison of the outcomes between the treatment and the comparator groups (Equation 1) (Concato et al., 2000). However, even adequately powered RCT may not always be feasible for reasons such as cost, time, ethical, and practical constraints (Sibbald and Roland, 1998). RCTs also rely on data collected on selected, homogeneous population under highly controlled conditions; hence, they provide evidence on efficacy rather than on effectiveness of interventions or treatments (Eichler et al., 2011).

(1) $$ATE=E[Y_{1}-Y_{0}]=E[Y_{1}]-E[Y_{0}]$$

With steadily increasing costs of health care and the introduction of novel, yet very expensive, pharmaceutical products and diagnostics, HTA agencies such as the UK National Institute for Health and Care Excellence (NCIE) are inquiring robust methods for evaluation of relative effectiveness and safety of medications, devices, and diagnostics in daily clinical practice. In contrast to efficacy, relative effectiveness of an intervention or treatment is “the extent to which an intervention does more good than harm, when compared to one or more alternative intervention(s)” when used under the routine setting of health care practice” (Eichler et al., 2011; Schneeweiss et al., 2011). In addition, for medical devices and diagnostics, waiting for evidence from RCTs when the health technology is diffusing in the clinical practice could be costly for the payers, inefficient from policy perspective, and methodologically questionable (Tarricone et al., 2016). On the other hand, regulators’ and HTA agencies’ perception of the importance of real-world data in complementing evidence on the relative effectiveness of health technologies has been steadily increasing (Makady et al., 2017; Yuan et al., 2018).

**Figure 1:**
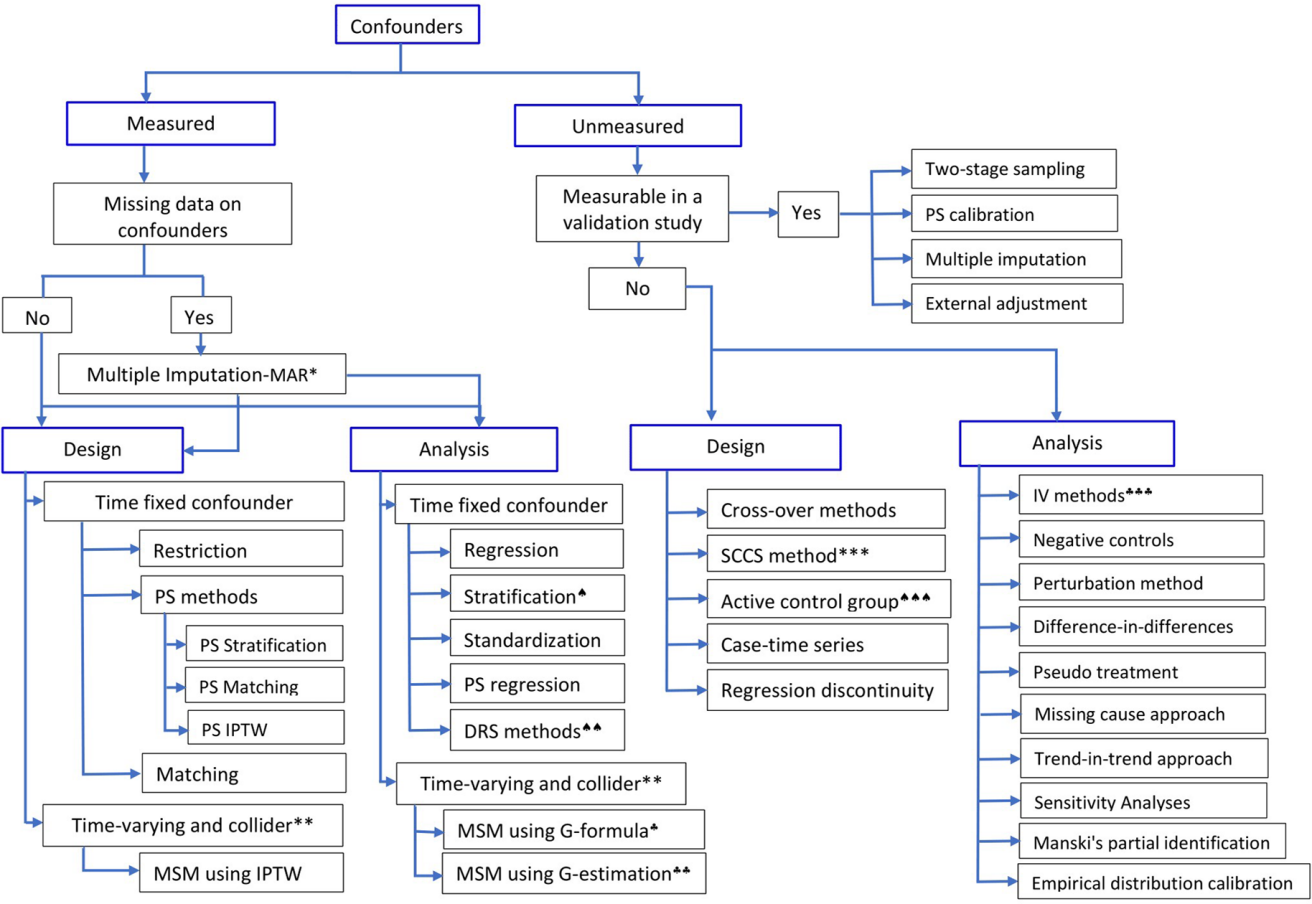
Methods to control for confounding in observational studies.*Multiple imputation is valid when the assumption of Missing at Random (MAR) holds;**if time-varying confounder is affected by previous treatment, all PS-based methods except marginal structural model (MSM) using inverse probability of treatment weight (IPTW) will give biased estimate;***self-controlled case-series design; ♠)stratification using effect modifier and adjustment within the strata to account for other covariates; ♠♠)Disease risk score (prognostic score) method; ♠♠♠)restriction or choosing an active comparison group vs non-user group; ♣)G-formula and ♣♣)G-estimation of structural nested models, which rely on specification of the outcome model; ♣♣♣)instrumental variable methods. (Adapted in part from Schneeweiss (2006), Uddin et al. (2016), and Zhang et al. (2018)).

The effect of a particular health technology, e.g., a medication, on a certain outcome event could also be investigated using non-randomized studies (i.e., observational or quasi-experimental) using routinely collected data (Schneeweiss et al., 2011, Ali et al., 2016, Bärnighausen et al., 2017). In observational studies, however, treatment selection is mainly influenced by the patient, the physician, and, to a certain extent, the health system characteristics. Hence, treated and untreated groups differ not only in receiving the treatment but also in other pretreatment characteristics, leading to non-comparability or non-exchangeability, a phenomenon leading to confounding bias (Greenland and Morgenstern, 2001). This means that differences in outcomes between the two groups, treated versus untreated, could be explained by either the treatment, or other pretreatment variables, or both. In other words, direct comparison of outcome events between the two groups leads to biased estimate of the treatment effect. Hence, any systematic difference in pretreatment characteristics between treatment should be accounted for by design, or analysis, or both (Rubin, 1997). Over the years, several methodologies have been developed to control for confounding bias in observational studies (**Figure 1**); the propensity score methods (Rosenbaum and Rubin, 1983) are among the popular approaches in pharmacoepidemiology and health technology evaluations (Ali et al., 2015).

Propensity score approaches were first introduced by Rosenbaum and Rubin in 1983 (Rosenbaum and Rubin, 1983), and their use to control for confounding has been increasing in the previous decade. Propensity score (PS) is a scalar summary of all measured pretreatment characteristics (often called potential confounders); stated formally, the propensity score e(X) is the conditional probability of receiving a certain treatment, versus a comparator or no treatment, given the measured pretreatment characteristics (Rosenbaum and Rubin, 1983), X, denoted as

(2) e(X)=pr(Z=1|X),

where Z = 1 for individuals in the treatment group and Z = 0 for individuals in the comparison group (Rosenbaum and Rubin, 1983; Rosenbaum and Rubin, 1984). Treated and untreated individuals with similar propensity scores have, on average, similar or comparable pretreatment characteristics, a situation similar to an RCT. However, this comparability, conditional on the propensity score, of the treatment groups is limited only to measured pretreatment characteristics included in the propensity score model and may not hold for unmeasured ones (Rosenbaum and Rubin, 1983). Hence, balancing these pretreatment potential confounders through propensity scores enables researchers to obtain a “quasi-randomization” of treatment groups to reduce confounding and hence to get a better estimate of the treatment effect. Implicitly, researchers assume “Strongly Ignorable Treatment Assignment” (SITA) given the measured covariates; this comprises “unconfoundedness” and “positivity” (Rosenbaum and Rubin, 1983). Unconfoundedness implies that all relevant pretreatment characteristics are measured and included in the propensity score model; hence, given these measured covariates are included in the propensity score, there is no unmeasured confounding. Positivity, on the other hand, implies that every individual has a non-zero (positive) probability of receiving all values of the treatment variable: 0 < *P*(*Z* = 1|*X*) < 1 for all values of Z (Rosenbaum and Rubin, 1983).

In the last decade, the propensity score methods have been popular among clinical researchers, their use in pharmacoepidemiology and HTAs has been ubiquitous, and they have undergone substantial methodological advances. On the other hand, confusions and misunderstandings on what a propensity score method can and cannot do as well as errors in the design, analysis, interpretation, and reporting of propensity score-based analyses are unfortunately all too common (Ali et al., 2015). With increasing availability of routinely collected electronic medical records for evaluation of effects (both comparative effectiveness and safety) of health technologies, and relatively rapid development of the methods, an up-to-date review of the methods and their characteristics is necessary. In this article, we aim to introduce propensity score methods with an emphasis on important aspects of the methods; describe their extended applications and recent developments; and discuss their strengths and limitations.

The manuscript, including the introduction, is organized into eight sections: the section *Introduction* has introduced RCT, observational studies, and propensity score in relation to HTA; the section *Variable Selection and Propensity Score Estimation* discusses variable selection and propensity score estimation approaches; the section *Covariate Balance Assessment* describes methods for assessment of covariate balance in propensity score methods; the section *Propensity Score Methods* summarizes the different types of propensity score methods; the section *Extended Applications* describes extended applications of propensity scores; the section *Advantages and Limitations of Propensity Score Methods* summarizes strengths and limitations of the propensity score methodology; the section *Reporting* highlights on reporting of propensity score based analysis; and the section *Conclusion* concludes the discussion.

## Variable Selection and Propensity Score Estimation

Observational studies using administrative or clinical databases often involve high dimensionality with respect to the number of pretreatment covariates available for analysis including socioeconomic characteristics, demographics, comorbidities, comedications, and health system characteristics, among others. The inclusion of a large number of covariates in conventional regression models, particularly in nonlinear models such as logistic regression and Cox regression models, requires sufficient number of outcome events (approximately 10 outcome events per covariate) (Peduzzi et al., 1995; Peduzzi et al., 1996; Cepeda et al., 2003). For example, to adjust for 5 confounders using logistic regression model, one would need to have 5*10 = 50 outcome events. However, many practical settings in pharmacoepidemiology and other HTAs involve relatively few or rare outcome events; hence, confounding adjustment using regression methods requires selection of a limited number of covariates to avoid problems such as over-fitting (Peduzzi et al., 1995). Alternatively, the use of propensity score methods to summarize a large pool of covariates into a single score, the propensity score, avoids over-fitting and collinearity issues in estimating treatment effects (Cepeda et al., 2003). When the number of covariates available in the study dataset is relatively small, it is common practice to include all the pretreatment covariates in the propensity score model; however, covariate selection might be required when researchers are presented with very large number (several hundreds) of covariates and limited number of outcome events (Schneeweiss et al., 2009).

Covariates selection in propensity score is often based on prior subject-matter knowledge on the relationships underlying the covariates in the study data, statistical tests on the association between the covariates and the outcome event (using p-values or change in effect estimates) (Brookhart et al., 2006; Patrick et al., 2011; Ali et al., 2015; Adelson et al., 2017), strength of associations with the treatment and/or the outcome event (Patrick et al., 2011; Ali et al., 2015; Adelson et al., 2017), and machine learning methods such as generalized boosted models (McCaffrey et al., 2004). Each approach has its own strengths and limitations; however, emphasis should be given to achieve balance on important prognostic pretreatment characteristics (Rosenbaum and Rubin, 1983) and not to improve model fit or to predict treatment as well as possible. Hence, the use of p-values, goodness-of-fit tests, and model discrimination tests such as c-statistics should be avoided (Weitzen et al., 2005; Patrick et al., 2011; Westreich et al., 2011). The iterative approach of model fitting, by including interactions and square terms of the covariates, and subsequent balance assessment, which was recommended in the seminal paper by Rosenbaum and Rubin (1983), is still a more robust approach. This application helps to achieve the goal of propensity score modelling, “improving balance” of potential confounders between treatment groups so that the groups are comparable or exchangeable conditional on the propensity score.

One of the greatest strengths of propensity score approaches is the separation of design from analysis, i.e., propensity score methods purposefully disregard outcome information at this stage of the design (Rubin, 2004b; Leacy and Stuart, 2014). That would also mean, as in the classical implementation of the methods, association between the covariates and the outcome event in the study data is not assessed for selection of covariates while constructing the propensity score model. However, this approach is not without disadvantages: failure to exclude colliders (variables that are common effects of the treatment and the outcome) and strong instruments (variables that are strongly related to treatment but independent of both the confounders and the outcome) can lead to increased bias in the estimated treatment effect (Pearl, 2011; Myers et al., 2011a, Myers et al., 2011b; Pearl, 2012; Ali et al., 2016).

It is important to emphasize that, similar to conventional regression modelling, intermediates (variables on the causal pathway between the treatment and the outcome) and colliders should not be included in the propensity score model (Greenland and Morgenstern, 2001) since including these variables will tend to increase (rather than reduce) bias. In addition, strong instruments should also be excluded, particularly when strong unmeasured confounding is a concern thereby avoiding any amplification of the residual bias (Pearl, 2011; Myers et al., 2011a; Myers et al., 2011b; Pearl, 2012; Ali et al., 2016). However, it is not common to come across with such a scenario; the use of propensity score method is meaningful when the assumption of “Strongly Ignorable Treatment Assignment”, SITA, is met (i.e., there is no unmeasured confounding given the measured covariates and also there is positivity) (Rosenbaum and Rubin, 1983). Compared to residual confounding by unmeasured characteristics, bias amplification should be considered a secondary concern; hence, researchers should be cautious and are advised to err on the side of including rather than excluding any potential confounder (Myers et al., 2011b; Ali et al., 2017c). Alternatively, when a strong instrument—essentially a proxy measure of difference in treatment—is identified that is independent of confounders and outcome, instrumental variable analysis can be a powerful tool to account for any unmeasured confounding (Angrist et al., 1996).

A common question asked by clinical researchers who have not used propensity score methods is “why do we need to estimate the probability that an individual receives a certain treatment versus a comparator while we certainly know from the data whether that particular individual has received the treatment?” A brief answer to this important question is as follows: propensity score exists both in RCT and in observational studies (Joffe and Rosenbaum, 1999; Rubin, 2004b; Ali et al., 2016). In RCT, the true propensity score is known by design or the treatment allocation mechanism, i.e., randomization. For example, consider a simple two-arm RCT in which individuals are assigned to a treatment versus a comparison group by flipping of a fair coin (also assume that the sample sizes are equal in both treatment groups). The propensity score for every individual, the probability of being assigned to the treatment group versus the comparator group, is equal to 0.5, apart from chance variations. In contrast, in observational studies, the true propensity score for individuals is unknown and is dependent on several pretreatment characteristics, both clinical and nonclinical, under consideration by the physician. As a result, the propensity score should be—and can often be—estimated using the study data (Joffe and Rosenbaum, 1999; Rubin, 2004b; D’Agostino, 2007; Ali et al., 2016). Estimation of the propensity score is needed to create a “quasi-randomized experiment” by using the individual’s probability of receiving the treatment as a summary score of all measured pretreatment covariates. It enables appropriate adjustment for measured potential confounders to estimate the effect of the treatment. This explains one of the key properties of the propensity score method: if we find two individuals with the same propensity score, one in the treated group and one in the untreated group, we can assume that these two individuals are more or less “randomly assigned” to one of the treatment groups in the sense of being equally likely to be treated or not, with respect to measured pretreatment characteristics (Ali et al., 2015; Ali et al., 2016).

In practice, the propensity score is often estimated using ordinary logistic regression model, in which treatment status is regressed on measured pretreatment characteristics (Austin, 2008a; Ali et al., 2015). The estimated propensity score is the predicted probability of receiving the treatment derived from the fitted logistic regression model. Logistic regression has several advantages: it is a familiar and well-understood statistical tool for researchers as well as easy to implement using standard statistical software packages (Setoguchi et al., 2008; Westreich et al., 2010; Ali et al., 2016). However, logistic regression is not the only approach; other methods have also been used including recursive partitioning (D’Agostino, 2007) and several machine learning methods, for example, classification and regression trees (CARTs), neural networks, and random forests (Setoguchi et al., 2008; Lee et al., 2010; Westreich et al., 2010; Lee et al., 2011). Comparative simulation studies favor the use of machine learning methods over logistic regression when there is moderate or high nonlinearity (square or cubic terms of covariates) and non-additivity (interactions between pretreatment covariates) in the propensity score models. This could be explained by the fact that machine learning methods include interactions and square terms by default (Setoguchi et al., 2008), compared to logistic regression where the researcher should “manually” include interactions and square terms. When important interaction and square terms are included, the performance of logistic regression is as good as other machine learning methods (Ali et al., 2017b).

## Covariate Balance Assessment

The aim of propensity score methods is to balance covariates between treatment groups and hence control for measured confounding (Rosenbaum and Rubin, 1983). Therefore, the quality of propensity score model should be assessed primarily on the covariate balance achieved. It should not be evaluated based on how well the propensity score model discriminates between treated and untreated individuals, i.e., whether the treatment assignment is correctly modeled (Rubin, 2004b; Westreich et al., 2011; Ali et al., 2015, Ali et al., 2016) or whether the subsequent estimates of treatment effect are smaller or larger than expected (Rosenbaum and Rubin, 1984; Hansen, 2004). Hence, propensity score modelling can be considered as an iterative step where the propensity score model is updated by adding different covariates, interactions between covariates, or higher-order terms of continuous covariates until an acceptable level of balance on important confounding variables is achieved (Rosenbaum and Rubin, 1984). It is also important to underline that variable selection and covariate balance are inseparably linked; however, covariate balance is often checked on a preselected list of pretreatment covariates (Ali et al., 2015). On the other hand, there are propensity score modelling techniques that optimize covariate balance while estimating the propensity score (Imai and Ratkovic, 2014; Austin, 2019).

It is helpful to start propensity score analysis by examining the distribution of propensity scores using histograms or density plots. This facilitates subjective judgment on whether there is sufficient overlap, also called “the common support,” between propensity score distributions of treated and untreated groups (Dehejia and Wahba, 2002). However, such plots should not be considered as proper measures of covariate balance; they can guide the choice of matching algorithms in propensity score matching and the number of strata in propensity score stratification (Ali et al., 2015; Ali et al., 2016). For example, when there is very little overlap in the propensity score distributions, matching treated and untreated individuals with replacement, with or without caliper, can be a better option because it will be challenging to find sufficient number of untreated individuals for the treated individuals (Ali et al., 2016). Inadequate overlap in the propensity score distributions, which can be quantified using overlapping coefficient (Ali et al., 2014), should also warn researchers that the dataset, no matter how large, could not support any causal conclusion about the effect of the treatment on the outcome of interest without relyng o untrustworthy model assumptions (Rubin, 1997; Ali et al., 2016).

To assess covariate-specific balance, several metrics have been proposed in the literature (Austin, 2009; Belitser et al., 2011; Ali et al., 2014). Each balance metric has its own advantages and limitations; the absolute standardized difference in means or proportions (ASMD) (Austin, 2009) is more robust in terms of sample size and covariate distribution requirements in comparison to other balance diagnostics, such as overlapping coefficients (Ali et al., 2014; Ali et al., 2015; Ali et al., 2016). The ASMD is also a familiar, easy-to-calculate and present, and well-understood statistical tool (Austin, 2009; Ali et al., 2015; Ali et al., 2016). Hence, it is recommended for checking and reporting covariate balances in propensity score methods (Austin, 2009; Belitser et al., 2011; Ali et al., 2014; Ali et al., 2015; Ali et al., 2016). The ASMD is calculated for each covariate and can be averaged to compute an overall covariate balance and to compare propensity score models (Belitser et al., 2011; Ali et al., 2014). The covariate-specific ASMD is useful to identify the variable that is still imbalanced and to modify the propensity score model with squares and interaction terms of the variable to improve its balance. Although there is no universal threshold below which the level of covariate imbalance is always acceptable (Imai and Van Dyk, 2004; Ali et al., 2016), the use of arbitrary cutoffs for balance diagnostics (e.g., < 10% for the ASMD) is common in the medical literature (Ali et al., 2015; Ali et al., 2016). Covariate balance is not only a property of the sample means but also of the overall distribution of the covariate; hence, higher-order sample moments of the covariate distribution such as variance should also be evaluated (Rosenbaum and Rubin, 1985; Rubin, 2001; Ho et al., 2007; Austin, 2009; Linden and Samuels, 2013). Rubin (2001) proposed the ratio of variances of treated and untreated groups as an additional check on balance; a variance ratio of 1.0 in the propensity score matched sample indicates a good matching and acceptable balance, and a variance ratio below 2 is generally considered acceptable balance (Rubin, 2001; Linden and Samuels, 2013).

In addition to numerical quantification of the covariate balance achieved by the specified propensity score model, graphical methods such as (weighted) side-by-side box plots, quintile-quintile (Q-Q) plots, plots of ASMD, and empirical density plots of continuous pretreatment covariates provide a simplified overview on whether balance on individual pretreatment covariates has improved, compared to pre-matching, pre-stratification, or pre-weighting (Rosenbaum and Rubin, 1983; Ali et al., 2016).

## Propensity Score Methods

Once the propensity score has been estimated, researchers have several options of using the propensity score in the design or analyses, including matching, stratification (also called subclassification), covariate adjustment using the propensity score, inverse probability of treatment weighting, and combinations of these methods (Rosenbaum and Rubin, 1983; Rosenbaum and Rubin, 1984; Rubin and Thomas, 2000; Hirano and Imbens, 2001; Johnson et al., 2018). Each method has its own advantages and disadvantages; the choice of a specific propensity score method is in part determined by the inferential goal of the research (i.e., the type of treatment effect estimand: the average treatment effect in the entire population, ATE, versus the average treatment effect in the treated population, ATT) (Imbens, 2000; Stuart, 2008; Ali et al., 2016). Although it is possible to estimate both ATT and ATE using all of the four propensity score methods, for example, by assigning different weights for the treated and untreated individuals, the default approach in each method might give slightly different estimand. For example, propensity score matching primarily estimates the treatment effect in the treated group, ATT (Imbens, 2004; Stuart, 2008). Therefore, to get an estimate of the average treatment effect in the entire population, ATE, one has to use either full matching (Hansen, 2004) or different weighting (Stuart, 2008, Stuart, 2010; Ali et al., 2015; Ali et al., 2016). The use of a specific propensity score method has also direct implication on the covariate balance assessment (Rosenbaum and Rubin, 1983; Rosenbaum and Rubin, 1984; Ali et al., 2016) and interpretation of the estimated treatment effect (Stuart, 2008; Ali et al., 2015; Ali et al., 2016).

### Propensity Score Matching

Propensity score matching, the most common application of propensity score (Ali et al., 2015), entails forming matched groups of treated and untreated individuals having a similar value of the propensity score (Rosenbaum and Rubin, 1983; Rubin and Thomas, 1996). The matching could be done in many ways: one-to-one or one-to-many (1:n, where n is the number of untreated individuals often up to five), exact or caliper matching, matching with or without replacement, stratified matching, and full matching (Hansen, 2004). However, one-to-one caliper matching without replacement is the most common implementation of propensity score matching (Ali et al., 2015; Ali et al., 2016). For detailed discussion on different matching approaches, we refer to the literature (Rosenbaum and Rubin, 1985; Hansen, 2004; Stuart, 2010).

Once a matched sample has been formed, covariate balance can be easily checked between the matched groups using one of the balance diagnostics, preferably ASMD, and then treatment effect can be estimated by directly comparing outcomes between treated and untreated individuals in the matched sample (Rosenbaum and Rubin, 1983; Rubin and Thomas, 1996). With dichotomous or binary outcomes such as the presence or absence of a disease (“Yes” or “No”), the effect of the treatment can be estimated as the difference or the ratio between the proportion of individuals experiencing the outcome event in each of the two treatment groups (treated vs. untreated) in the matched sample. If the outcome is continuous, for example blood pressure measurement or HBA1c level, the effect of the treatment is estimated as the difference between the mean outcome for treated and the mean outcome for untreated individuals in the matched sample (Rosenbaum and Rubin, 1983).

If matching is done with replacement or in one-to-many matching, weights should be incorporated to account for the multiple use of the same untreated individual to match with several treated individuals or the multiple use of the same treated individual to match with several untreated individuals, respectively (Stuart, 2010). Whether or not to account for the matched nature of the data in estimating the variance of the treatment effect, for example, using paired t-test for continuous outcome or McNemar’s test for binary outcome, is an ongoing discussion (Schafer and Kang, 2008; Stuart, 2008; Austin, 2008a; Austin, 2011).

The most appealing feature of propensity score matching is that the analysis can partly mimic that of an RCT, meaning that the distribution of measured pretreatment covariates will be, on average, similar between treatment groups. Hence, direct comparison of outcomes between treated and untreated groups within the propensity score matched sample has the potential to give unbiased estimate of the treatment effect, depending on the extent to which the measured variables have captured the potential confounding factors (Rosenbaum and Rubin, 1983). However, RCT, on average, guarantees balance on both measured an unmeasured confounders, whereas propensity score improves balance on measured confounders but those of unmeasured confounders only to the extent that they are related to the measured confounders included in the propensity score model (Rubin, 2004b; Austin, 2011). Other useful features include: separation of the design from analysis *via* preprocessing of the data to improve covariate balance without using outcome data, thereby a minimal reliance on model specification; relatively easy assessment, visualization, and communication of covariate balance using simple statistics or plots; and qualitative indication of whether the dataset at hand is good enough to address the causal question without relying on untrustworthy “model-dependent” extrapolations (Rubin, 2004b; Ho et al., 2007; Ali et al., 2016).

Recently, the use of propensity score for matching has been criticized on the basis of an argument that propensity score matching approximates complete randomization and not completely blocked randomization; hence, it engages in random pruning or exclusion of individuals during matching. “Unlike completely blocked randomization, random exclusion of individuals in propensity score matching, as in complete randomization, means a decrease in sample size leading to covariate imbalance and more model dependence, so called the ‘propensity score paradox’” (King and Nielsen, 2016). At first this might seem a valid argument; however, the practical implication of this paradox is very limited, if any (Ali et al., 2017a). This is partly due to the fact that propensity score matching could do better than complete randomization with respect to the balance of measured covariates if variables related to treatment are included in the propensity score model (Joffe and Rosenbaum, 1999). In addition, the use of matching algorithms such as caliper matching or matching with replacement retains the best matches thereby avoiding random pruning or exclusion, and hence the paradox is not a big concern. Furthermore, it is currently a standard practice to check covariate balance in the propensity score matched sample before estimating the treatment effect, further minimizing any risk of exacerbating covariate imbalance (Ali et al., 2015).

Similar to RCT, when there are residual differences in pretreatment characteristics between treatment groups in propensity score matched sample, regression adjustment can be used on the matched sample to reduce bias due to residual differences in important prognostic factors (Rubin and Thomas, 2000; Imai and Van Dyk, 2004; Schafer and Kang, 2008). This method has been described as a doubly robust (DR) approach, i.e., correct specification of either the matching or the regression adjustment, but not necessarily both, is required to obtain unbiased estimate of the treatment effect (Schafer and Kang, 2008; Funk et al., 2011; Nguyen et al., 2017). Propensity score matching primarily estimates the effect of treatment in the treated individuals (ATT), not the effect of treatment in the population (treated and untreated individuals, ATE) (Imbens, 2004; Stuart, 2008). This is because the closest untreated and treated individuals are matched and the remaining untreated individuals that were not matched are often excluded from the analysis (Stuart, 2008; Ali et al., 2016). It is important to emphasize that exclusion of unmatched individuals from the analysis not only affects the precision of the treatment effect estimate but also could have consequences for the generalizability of the findings, even for the ATT (Lunt, 2013; Ali et al., 2016). For example, exclusion of treated individuals due to a lack of closer untreated matches could change the estimand from the effect of treatment in the treated (ATT) to the effect of treatment in those treated individuals for whom we can find untreated matches (ATT) (Lunt, 2013; Ali et al., 2016). However, it is possible to estimate the ATE in the matched sample with slight modifications of the matching algorithms. For example, using full matching that retains all the treated and untreated individuals in the study data, one can estimate either the ATE or ATT (Hansen, 2004; Stuart, 2010). Generally, matching discards some data (often unmatched untreated individuals); however, it may increase the efficiency, reducing the estimated standard error, of the treatment effect estimate by reducing heterogeneity of observations (Ho et al., 2007; Ali et al., 2016).

### Propensity Score Stratification

Propensity score stratification, also called propensity score subclassification, involves grouping individuals into strata based on their propensity scores (often 5 groups using quintiles or 10 groups using percentiles). Within these strata, treated and untreated individuals will have a similar distribution of measured covariates; hence, the effect of the treatment can be estimated by direct comparison of outcomes between treated and untreated groups within each strata (Rosenbaum and Rubin, 1984; D’Agostino, 2007; Ali et al., 2017a). The stratum-specific treatment effects can then be aggregated across subclasses to obtain an overall measure of the treatment effect (Rosenbaum and Rubin, 1984).

Rosenbaum and Rubin (1983, 1984) proposed quintile stratification on the propensity score based on their finding that five equal-size propensity score strata removed over 90% of the bias due to each of the pretreatment covariates used to construct the propensity score. However, it is recommended that researchers examine the sensitivity of their results to the number of subclasses by repeating the analysis using different quantiles of the propensity score (Imai and Van Dyk, 2004; Adelson et al., 2017). Similar to matching, residual imbalances after stratification can be accounted for using regression adjustment within each stratum (Rosenbaum and Rubin, 1984; Rubin, 2001). Alternatively, the propensity score, defined as quintiles and deciles, can be used as a categorical variable in a model-based adjustment to estimate treatment effects (Rosenbaum and Rubin, 1984; Ali et al., 2016).

Propensity score stratification can estimate the stratum-specific ATT, or the overall ATT across strata, or the ATE, depending on how the subclass treatment effect estimates are weighted. Weighting stratum-specific estimates by the total number of individuals (treated and untreated) in each stratum yields the ATE. On the other hand, weighting stratum-specific estimates by the proportion of treated individuals in each stratum provides ATT (Stuart, 2010; Ali et al., 2016). Similarly, pooling stratum-specific variances provides pooled estimates of the variance for the pooled ATT or ATE estimate (Imbens, 2004; Ali et al., 2016). Pooling the stratum-specific treatment effect is straightforward when treatment effect is homogeneous among the propensity score strata (Ali et al., 2016). When there is heterogeneity of treatment effect among the strata even after automated iterations of the number and boundaries of propensity score strata (Imbens, 2004; Imbens and Rubin, 2015; Ali et al., 2016), pooling the stratum-specific treatment effect might complicate interpretation of the treatment effect estimate (Ali et al., 2014; Ali et al., 2016). In the presence of treatment effect modification regardless of the presence of confounding, Mantel-Haenszel methods do not estimate a population parameter (ATE); hence, estimating the effect of treatment in the treated (ATT) rather than the whole population (ATE), for example, using propensity score matching is preferable (Stürmer et al., 2006b). Alternatively, one could standardize the stratum-specific estimates to a specified distribution of propensity scores, for example, to calculate a standardized mortality ratio (AMR) from the stratum-specific estimates (Stürmer et al., 2006b; Lunt et al., 2009)

Stratification has several advantages: it is an easy and well-understood method to implement; it is straightforward to evaluate and communicate covariate balance, and to interpret particularly to non-technical audiences; it separates the design of the study from the analysis, like propensity score matching, hence less dependent on parametric models (Rosenbaum and Rubin, 1984); it is less sensitive to nonlinearities in the relationship between propensity scores and outcomes; and it can accommodate additional model-based adjustments (Rosenbaum and Rubin, 1983; Rosenbaum and Rubin, 1984). However, this propensity score approach is prone to residual confounding, which might be an issue due to propensity score heterogeneity within the strata.

### Regression Adjustment Using Propensity Score

The propensity score, as a single summary of all covariates included in the propensity score model, can be included as a covariate in a regression model of the treatment, i.e., the outcome variable is regressed on the treatment variable and the estimated propensity score (Rosenbaum and Rubin, 1983; Ali et al., 2016). Although this approach is very easy to implement, it is generally considered to be a sub-optimal application of the propensity score for several reasons: 1) The treatment effect estimation is highly model-dependent because it mixes the study design and data analysis steps; hence, it requires correct specification of the propensity score model (Rubin, 2004b; Johnson et al., 2018). 2) It also makes additional assumptions unique to regression adjustment; the relationship between the estimated propensity score and the outcome must be linear and there should be no interaction between treatment status and the propensity score (Rosenbaum and Rubin, 1983; Austin, 2011; Ali et al., 2016). However, both assumptions can be checked with the data, and can be relaxed if necessary, for example, by combining with propensity score stratification. 3) It enables estimation of the ATE; however, its interpretation is complicated particularly in nonlinear models such as logistic regression or Cox regression where the estimand of interest is non-collapsible. Non-collapsibility refers to a phenomenon in which, in the presence of a non-null treatment effect, the marginal (overall) treatment effect estimate is different from the conditional (stratum-specific) treatment effect estimate, even in the absence of confounding (Greenland et al., 1999; Austin, 2008b). In addition, assessment and communication of covariate balance are not straightforward (Ali et al., 2016).

### Inverse Probability Treatment Weighting

Inverse probability weights (IPW) calculated from propensity score can also be used to create a weighted “artificial” population, also called a “pseudo-population” in which treatment and measured pretreatment characteristics included in the propensity score are independent (Hernán et al., 2000; Robins et al., 2000; Cole and Hernán, 2008; Ali et al., 2016). Hence, treated individuals will be assigned weights equal to the inverse of their propensity scores (1/PS, as they have received the treatment) and untreated individuals will be assigned weights equal to the inverse of one minus their propensity scores [1/(1 – PS)] (D’Agostino, 2007). A particular diagnostic concern in using propensity score weighting is that individuals with extremely large weights may disproportionately influence results and yield estimates with high variance (Lee et al., 2011). When some individuals have probabilities of receiving the treatment close to 0 or 1, the weights for such individuals become extremely high or extremely low, respectively (Ali et al., 2016). Weight stabilization to “normalize” the range of the inverse probabilities is often considered: the “1” in the numerator of the inverse probability weights can be replaced with the proportion of treated individuals and the proportion of untreated individuals for treated and untreated individuals, respectively (Hernán et al., 2000; Ali et al., 2016).

Alternative approaches such as weight trimming and weight truncation have been suggested (Cole and Hernán, 2008; Lee et al., 2011). Weight trimming involves removing individuals in the tails of the propensity score distributions using percentile cut-points (Cole and Hernán, 2008; Lee et al., 2011), i.e., individuals who have extreme values of the propensity score—both very high and very low are excluded. On the other hand, weight truncation involves setting a maximum allowable weight, W*_ma_*), such that individuals with a weight greater than W*_ma_*) will be assigned W*_ma_*) instead of their actual weights. Both approaches may help stabilize weights, reduce the impact of extreme observations, and can improve the accuracy and precision of parameter estimates; however, both involve bias-variance trade-offs (Lee et al., 2011). For example, trimming the tails excludes some individuals with extreme values and hence changes the population, which might introduce bias depending on the cut-off (Cole and Hernán, 2008). Recently, Li et al. (2018) suggested a different set of weights called “overlapping weights” which weight each individual proportional to its probability of receiving the alternative treatment, i.e., the overlap weight is defined as 1-PS for a treated individual and PS for an untreated individual. Unlike standard IPW, the overlap weights are bounded between 0 and 1; hence, they are less sensitive to extreme weights. It also means that there is no need for arbitrary choice of a cut-off for inclusion in the analysis as well as exclusion of individuals, unlike weight trimming (Li et al., 2018).

In the weighted population, weighted standardized difference can be used to compare means, proportions, higher-order moments, and interactions between treated and untreated individuals. In addition, graphical methods can be employed to compare the distribution of continuous covariates between treated and untreated individuals (Austin and Stuart, 2015). Once sufficient covariate balance is achieved, the effect of the treatment can be estimated by direct comparison of outcomes between treated and untreated groups. The weights can also be used in weighted regression models to estimate the effect of the treatment; and adjustment can be made for covariates that are not sufficiently balanced in the weighted sample. This method focuses on estimating the average treatment effect in the entire population (ATE); modification of the weights allows to estimate the average treatment effect in the treated population (ATT) (Stuart, 2010; Ali et al., 2016). Most importantly, the variance estimation should take into account the weighted nature of the “pseudo-population” since some observations can have weights that are unequal to one another (hence, potentially inducing a within-individual correlation in outcomes), for example, by using the sample weights in robust variance estimation (Hernán et al., 2000; Cole and Hernán, 2008; Austin and Stuart, 2015). Alternatively, bootstrapping could be used to construct 95% confidence intervals, which also takes into account the estimation of the propensity score, in addition to the lack of independence between duplicate observations in the weighted sample (Hernán et al., 2000; Austin and Stuart, 2015; Ali et al., 2016; Ali et al., 2017b).

**Figure 2:**
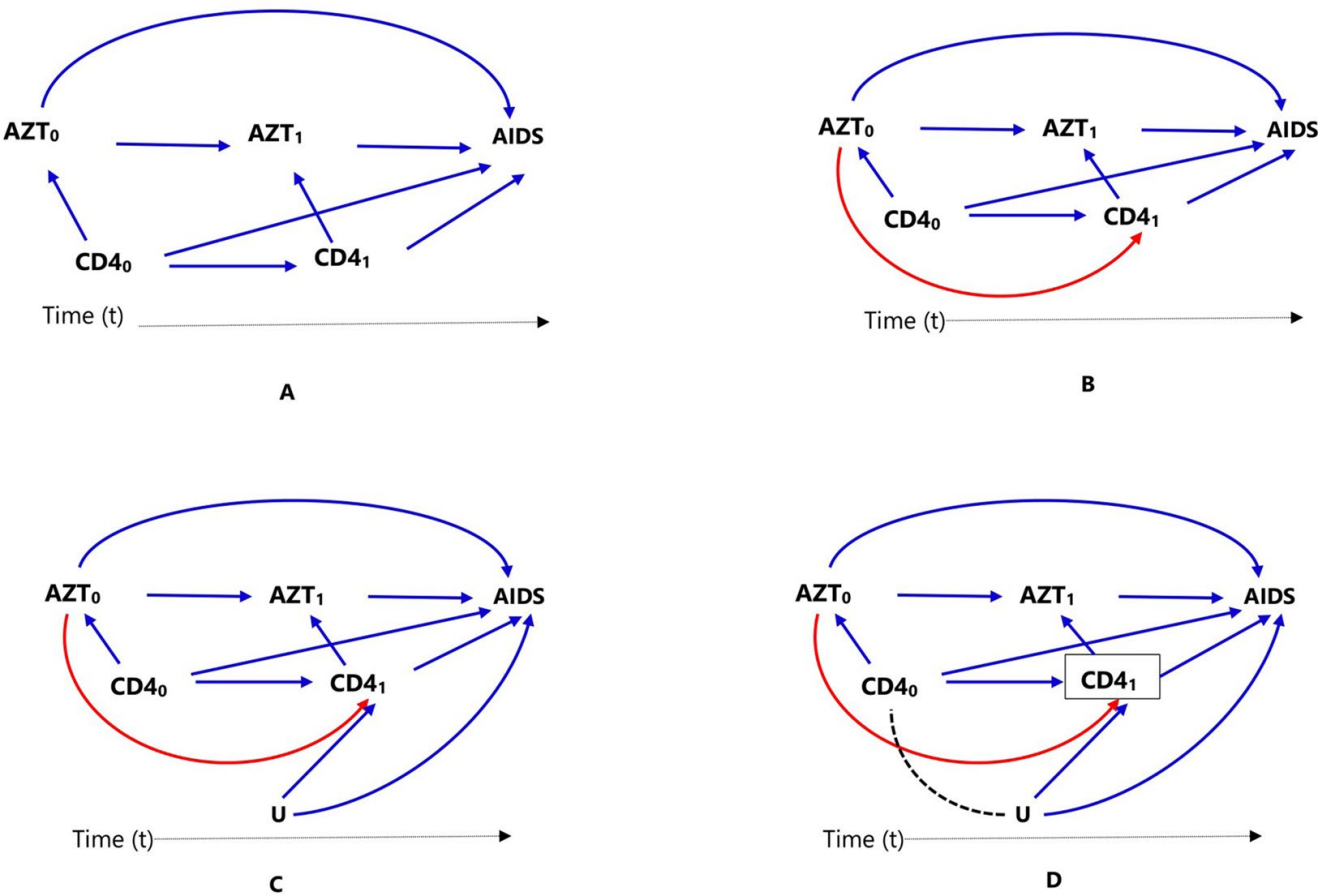
Causal diagrams representing time-varying treatment (AZT), outcome (progression to AIDS, AIDS), and time-varying confounding (CD4 count). Time-varying confounding is not affected by prior treatment **(A)**, time-varying confounding is affected by prior treatment **(B)**, time-varying confounding affected by unmeasured factor U, which is also associated with the outcome **(C)**, and conditioning or stratifying on time-varying confounder, indicated by box around CD41), creates association between time-varying confounder CD4_0_) and unmeasured factor U **(D)**.

Inverse probability of treatment weights (IPTW) can be also be used to estimate parameters of marginal structural models (MSMs) to deal with time-varying confounding (Hernán et al., 2000), time-modified confounding (Platt et al., 2009), and competing risks (Hernán et al., 2000; Ali et al., 2017b). Hence, the implementation of propensity scores as inverse probability weights is often referred to as MSM using IPTW. All other propensity score approaches can only be extended to time-varying confounding and treatment settings under certain conditions as described in **Figure 2**. Comparison of the four propensity score approaches is summarized in **Table 1**.

**Table 1:** Comparison of the different propensity score methods.

Characteristics	Matching^a^	Stratification^b^	Regression^c^	IPTW^d^
Model dependence	Minimum	Minimum	High	Minimum
Application^1^	Easy	Easy	Easy	Complex
Overall transparency	High	High	Low	Medium
Easy to communicate	Yes	Yes	Not always	Not always
Design and analysis	Separated	Separated	Mixed	Separated
Easy to check balance	Yes	Yes	No	Yes
Requires unique assumption^2^	No	No	Yes	No
Excluded individuals from analysis^3^	Yes	No	No	Yes-No
Variance estimation	Not clear	Easy	Easy	Complex
Easy to interpret^4^	Not always	Yes	No	Often
”Propensity score paradox”	Sensitive	No	No	No
Estimand^5^	Often ATT	ATE, ATT	ATE	ATE, ATT
Time-varying confounding^6^	No	No	No	Yes
Multiple treatments	Possible	Complex	Complex	Easier
Multi-level treatment applications	Exist	Exist	None	Exist
Treatment effect modification	Easier	Complex	Easier	Complex

^a^Constructs treated and untreated matched groups with similar propensity scores. ^b^Constructs subgroups of treated and untreated individuals, often quintiles or deciles of PS. ^c^PS is used, as a single summary of all covariates included in PS model, in regression model. ^d^PSs are used as weights to create a pseudo-population in which exposure and measured covariates included in the treatment (PS) model are independent (Ali et al., 2016). ^1^Estimation of stabilized weights as well as extension to time-varying treatment and confounding setting in MSMs framework can be complex (Ali et al., 2016). ^2^Requires correct specification of PS and outcome model, apart from the basic assumptions that there is positivity and no unmeasured confounding (Ali et al., 2016). ^3^Weight trimming excludes some individuals in the tails of the propensity score distribution. ^4^In PSM, when treated individuals are excluded, interpretation of the treatment effect may change, not just ATT and in Stratification, when there is treatment effect modification by the PS, in regression adjustment using PS, when non-collapsible effect measures such as odds ratios are used. ^5^Modification of the matching or weighting method enable to estimate either ATT or ATE. ^6^When time-varying confounder is affected by previous treatment, all the propensity score based methods fail to correctly control for the confounding bias including standard IPWs; however, MSMs using IPWs.

## Extended Applications

### Time-Varying Treatments

In clinical practice, it is common for patients to start on a certain medication, stop or switch to another one (for example, due to intolerance or lack of adequate response); in such cases, treatment might be treated as a time-varying exposure. Consider a cohort study to estimate the effect of antiretroviral zidovudine treatment (AZT) in HIV (human immunodeficiency virus) positive individuals, on progression to AIDS (acquired immune deficiency syndrome), where CD4 count is a confounder. Assuming individuals show up for clinical visits at baseline/pretreatment (t = 0) and then every 6 months (t = 1, 2, 3,…), and CD4 counts are recorded at these visits (CD4*_t_*), represented as CD4_0_, CD4_1_, CD4_2_,…). If AZT is a time-varying dichotomous treatment variable indicating whether the individual is on antiretroviral treatment at each of the visits (AZT*_t_*, represented as AZT_0_, AZT_1_, AZT_2_,…), this means, an individual’s treatment plan, at each subsequent visit (t = 1,2,…), is time-varying: the clinician in consultation with the individual decides treatment AZT*_t_* based on the changing values of the individual’s clinical and demographic history recorded during the previous and current visits. These include prior treatment history, current CD4 count, and other confounders, which are not included in this discussion and ignored for now for the sake of simplicity. The relationships between treatment, confounder, and outcome are presented using directed acyclic graphs (DAGs) for clarity.

In **Figure 2**, we considered two time points or visits t = 0 (baseline/pretreatment) and t = 1; hence, CD4_0_ refers to baseline CD4 count and AZT_0_ refers to treatment at the first visit. Treatment decision at the first visit AZT_0_ is influenced by pretreatment CD4 count (CD4_0_), represented in **Figure 2A** by the arrow from CD4_0_ to AZT_0_. In the second visit (t = 1), treatment decision AZT_1_ is based on previous treatment (AZT_0_) and CD4 count at the current visit (CD4_1_), represented in **Figure 2A** by the arrows from AZT_0_ and CD4_1_ to AZT_1_.

In settings such as DAG of **Figure 2A**, where there is no arrow from AZT_0_ to CD4_1_ implying previous treatment does not affect current CD4 count, all the standard propensity score approaches can deal with the time-varying confounder CD4 count by matching, conditioning, stratification, or weighting, for example, by combining with time-varying Cox models to estimate the treatment effect. However, this is not biologically plausible; RCTs have proved that antiretroviral treatment indeed affects CD4 count. It is important to mention that there are many practical examples where both treatment and confounders are time-varying or dynamic, but previous treatment does not affect time-varying confounder; hence, the DAG in **Figure 2A** may still be valid in other situations.

When a time-varying confounder (such as CD4 count in our example, CD4_1_) is affected by previous treatment (AZT_0_) as in the DAG of **Figure 2B**, the time-varying confounder (CD4_1_) is also an “intermediate” for the effect of previous treatment (AZT_0_) on the outcome (progression to AIDS), represented by the path AZT_0_ → CD4_1_ → AIDS. Furthermore, if there is an unmeasured common cause (U) of both the time-varying confounder (CD4_1_) and the outcome (progression to AIDS) as in DAG of **Figure 2C**, the time-varying confounder (CD4_1_) is also a “collider” on the path AZT_0_ → CD4_1_ ← U → AIDS (the arrows from U and CD4_0_ collide on CD4_1_). Hence, the path AZT_0_ → CD4_1_ ← U → AIDS is a closed or non-causal path because it is blocked at CD4_1_ (using DAG terminologies). It also means that there is no association between AZT_0_ and U unless one conditions, matches, or stratifies on this collider, CD4_1_ (Hernán et al., 2000; Robins et al., 2000). Such a time-dependent variable is a confounder, an intermediate, and also a collider all at the same time; hence, adjustment requires careful consideration.

Conventional statistical approaches including propensity score methods (matching, stratification, and regression adjustment) that condition or stratify on such a covariate will result in a biased estimate of the treatment effect (Hernán et al., 2000; Robins et al., 2000). This happens because conditioning or stratifying on an intermediate will adjust away the indirect effect of the treatment mediated by the cofounder, in this case CD4_1_; and conditioning or stratifying on a collider creates a spurious association between the treatment and the unmeasured common cause that did not exist before conditioning (creating an open backdoor path AZT_0_ → CD4_1_,… U→ AIDS), which is indicated by using dotted lines in the DAG of **Figure 2D**, leading to collider-stratification bias (Hernán et al., 2000; Cole et al., 2009; Ali et al., 2013).

In such settings, MSM using inverse probability weighting is the method of choice; unlike conditioning or stratification, weighting creates a “pseudo-population” in which the association between the time-varying confounder and treatment is removed (Hernán et al., 2000; Robins et al., 2000). Additional methods are also available to deal with time-varying treatment and confounding including other classes of marginal structural models (g-formula and g-estimation of structural nested models) (Hernán et al., 2000; Robins et al., 2000).

It is straightforward to hypothesize that such a time-varying confounding can also be time-modified, which means not only the confounder (CD4 count) change over time but also its association with the treatment and its impact on the outcome (progression to AIDS) varies during these times. The effects of the confounder change over time mean that the strength of association between CD4_0_ and AIDS (CD4_0_ → AIDS) is different from that of CD4_1_ and AIDS (CD4_1_ → AIDS) (Platt et al., 2009). However, time-modified confounding might still exist in longitudinal treatment settings where the confounder is time-invariant or fixed. Standard methods are sufficient to deal with time-modified confounding unless the confounder is both time-varying and affected by previous treatment, which requires the implementation of marginal structural models, such as using inverse probability weighting.

### Multiple Treatments

Propensity score methods are often used to estimate the effect of a binary treatment (whether treatment is received: Yes = 1 or No = 0) in observational data. However, with more than two levels of treatment, which is common in pharmacoepidemiology such as comparison of three or more statins (e.g., simvastatin, atorvastatin, fluvastatin, lovastatin, pravastatin, and rosuvastatin) or of multiple doses of a certain medication (e.g., low, medium and high doses), estimation of treatment effects requires additional assumptions and modelling techniques (Imbens, 2000; McCaffrey et al., 2004). These include the use of multinomial logistic and multinomial probit models for nominal treatments and ordinal logistic regression or the proportional odds model for ordinal treatments (Imbens, 2000). Alternatively, generalized boosted model, a machine learning approach involving an iterative process using multiple regression trees to capture complex, nonlinear, and non-additive relationships between treatment assignment and pretreatment covariates without the risk of over-fitting the data, can be used to fit inverse probability weighting for multiple treatments (McCaffrey et al., 2004). However, applications in pharmacoepidemiology using observational data are infrequent partly due to methodological complexities in fitting the models and understanding their assumptions as well as limited availability of guidance documents on these methods.

### Multilevel Treatments

Propensity score methods have been extensively studied and widely applied in a single-level treatment (no clustering among participants); however, most healthcare data have a multilevel structure such that individuals are grouped into clusters such as geographical areas, treatment centers (hospital or physician), or insurance plans (Goldstein et al., 2002). The unknown mechanisms that assign individuals to clusters may be associated with individual-level measured confounders (such as race, age, and clinical characteristics) and unmeasured confounders (such as unmeasured severity of disease, aggressiveness in seeking treatment) (Li et al., 2013). These measured and unmeasured confounders might also create a cluster-level variation in treatment and/or outcome. If this variation is correlated with group assignment at the group or cluster level, it might lead to confounding (Greenland, 2000; Li et al., 2013). Hence, the use of standard regression or propensity score methods ignoring the cluster structure should be avoided. This is because ignoring the cluster structure often leads to invalid inferences: not only the standard errors are inaccurate but also the cluster-level effects could be confounded with individual-level effects.

Propensity score matching and weighting are often used in such settings (Arpino and Mealli, 2011; Li et al., 2013). One might consider the use of within-cluster PSM (of treated and untreated individuals), which automatically achieves perfect balance on all the measured cluster characteristics. However, it is very unlikely, particularly in small clusters, to find a sufficient number of untreated matches to treated individuals in the same cluster. Alternatively, PSM could be performed across clusters taking into account the cluster structure in the propensity score estimation model. Preferably, cluster structure should be taken into account in estimation of both the propensity score and the treatment effect (Li et al., 2013).

Multilevel regression models that include fixed effects and/or random effects have been developed (Greenland, 2000; Goldstein et al., 2002), and extended to propensity scores approaches (Arpino and Mealli, 2011). Empirical applications of such methods in medication and device effectiveness and safety are rare. However, simulations studies have shown that multilevel propensity score matching (Arpino and Mealli, 2011) and weighting approaches (Li et al., 2013), without imposing a within-cluster matching or weighting requirement, reduce bias due to unmeasured cluster-level confounders.

### Missing Data

Missing data is a common problem in the estimation of treatment effects using routinely collected data. The impact of such missing data on the results of the treatment effect estimation depends on the mechanism that caused the data to be missing and the way missing data are handled. Missing data can be categorized into three distinct classes based on the relationship between the missing data mechanism and the missing and observed values: i) Missing Completely at Random (MCAR), when the missing data mechanism is unrelated to the values of any variable, whether missing or observed. Hence, the observed values are representative of the entire sample without missing values. ii) Missing at Random (MAR), when the missing data mechanism is unrelated to the missing values but may be related to the observed values of other variables. iii) Missing Not at Random (MNAR), when the missing data mechanism is related not only to the observed values of other variables but also to the missing values (Rubin, 1996). For each of the missing data patterns, different statistical techniques are used to correct for its impact on the quality of the inference. It is important to emphasize that MCAR, MAR, and MNAR could exist for different variables in a specific data. However, if one variable is MAR or MNAR, generally, the dataset is considered MAR or MNAR, respectively.

Complete case analysis, including only those individuals who have no missing data in any of the variables that are required for the analysis, performs well when data are MCAR and may be valid under some MAR and MNAR conditions. However, it often results in biased estimate of the treatment effect if missing is at random (MAR) (Rubin, 1996; Sterne et al., 2009). In MAR, as stated before, any systematic difference between the missing values of a variable and the observed values of the variable can be explained by differences in observed data (Sterne et al., 2009). Furthermore, missing data in several variables often lead to exclusion of a substantial proportion of the original sample, which leads to a substantial loss of precision (i.e., power) and hence estimates with wider confidence intervals (Cummings, 2013). Other approaches to deal with missing data include: 1) replacing missing values with values imputed from the observed data (for example, using the mean of the observed values); 2) using a missing category indicator; and 3) using the last observed value to replace missing values particularly in longitudinal studies [often called “last observation carried forward” (LOCF)]. These three approaches are generally statistically invalid, except under certain conditions, and they might lead to serious bias (Rubin, 1996; Sterne et al., 2009). Missing category indicator and LOCF approaches require specific assumptions for validity that are distinct from the MCAR, MAR, and MNAR categorization. On the other hand, single imputation of missing values (mean imputation) usually results in too small standard errors, because it fails to account for the uncertainty about the missing values (Sterne et al., 2009).

**Figure 3:**
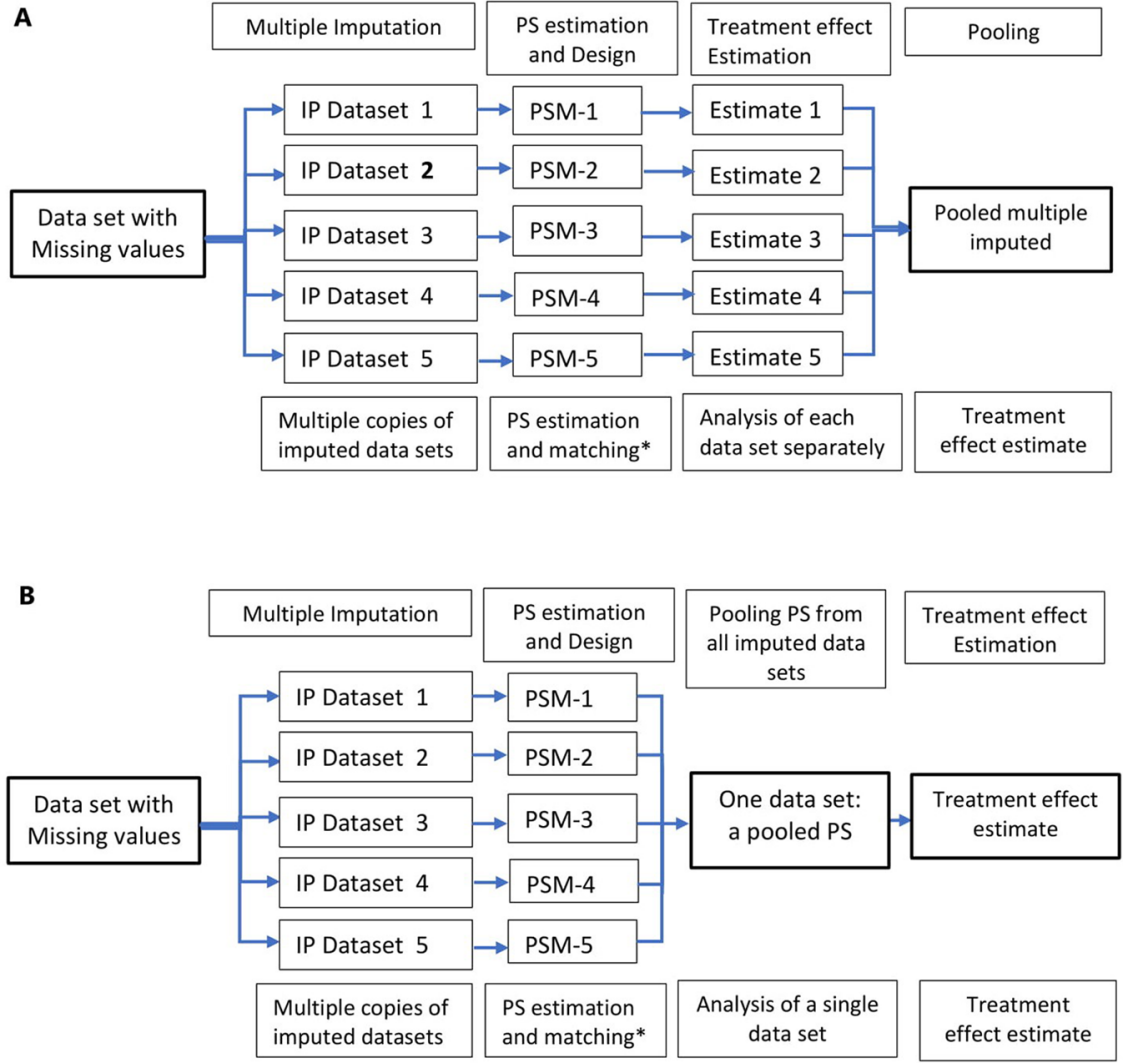
Multiple imputation in propensity score methods; multiple copies of imputed data are created and propensity score is estimated using these datasets. Treatment effects are estimated in several datasets **(A)** and propensity scores from multiple datasets are pooled and treatment effect estimated in a single dataset **(B)**. *Other PS methods, stratification, IPTW, and covariate adjustment using PS could also be used instead of matching.

A relatively flexible approach to allow for the uncertainty in the missing data is multiple imputation. Multiple imputation involves creating multiple different copies of the dataset with the missing values replaced by imputed values (Step 1); estimating treatment effects in each copy of the data (Step 2); averaging the estimated treatment effects to give overall estimated measure of association and calculating standard errors using Rubin’s rules (Step 3) (Rubin, 1996; Rubin, 2004a). Applications of propensity score methods in data with missing values involve a similar approach: 1) creation of multiple copies of imputed data; 2) estimation of propensity scores and treatment effects in each of the imputed copies of the dataset (Qu and Lipkovich, 2009; Leyrat et al., 2019); and 3) pooling of treatment effects by averaging across the multiple datasets and estimation of standard errors using Ruben’s rule (Crowe et al., 2010; Leyrat et al., 2019) (**Figure 3A**). An alternative approach is pooling the propensity scores from the multiple copies of data, in step 2, and conducting the analysis in the pooled data (**Figure 3B**); however, this method has been proved sub-optimal in terms of bias reduction (Leyrat et al., 2019).

## Advantages and Limitations of Propensity Score Methods

Previous literature reviews of observational studies have found that results from both traditional regression and propensity scores analyses are similar (Shah et al., 2005; Stürmer et al., 2006a). These findings may be in part due to sub-optimal implementations of propensity score methods (Shah et al., 2005; Austin, 2008a; Ali et al., 2015); however, similarity of findings has been used to question the need for propensity score methods if they do not provide better ways to improve confounding control. Despite these findings, propensity score methods will remain advantageous for several reasons compared to covariate-adjustment techniques, which correct for covariate imbalances between treatment groups by conditioning them in the regression model for the outcome.

### Transparency

Propensity score methods primarily aim at balancing treatment groups with respect to covariate distributions; when sufficient covariate balance is achieved, it is relatively easy to check and communicate the balance (Ali et al., 2015, Ali et al., 2016) by using simple graphical tools or quantitative statistics. In addition, propensity score methods, unlike regression adjustment, can give investigators an insight into the quality of the data at hand. Inadequate overlap in propensity score distributions (also called poor “common support”) between treatment groups should be considered as a warning that the data set at hand may not be sufficient to reliably address the causal question without “model-dependent” extrapolations based on untrustworthy assumptions (Dehejia and Wahba, 2002; Rubin, 2004b; Rubin, 2007; Ali et al., 2016). In some cases, the researcher might decide to focus on individuals only in the overlapping regions using propensity score matching or trimming; as a consequence, the conclusions of the findings should be restricted to individuals that are sufficiently represented in the overlapping regions of the propensity score distributions (Ali et al., 2016). Conventional regression methods do not provide the researcher with these possibilities. Furthermore, covariate balance in regression methods is a “black-box” and, irrespective of inadequate overlap (i.e., when the treated and untreated groups are disparate on pretreatment covariates), conventional models use extrapolations to estimate treatment effects that may not be generalizable to the entire population in the data set.

### Design Tools

Similar to RCTs, propensity score methods can be considered as design tools for pre-processing of the data (matching, stratification, and weighting) without using any outcome information at this stage. As a result, formal causal inference models (also called the potential outcomes framework) (Rubin, 2005) can be applied to clearly specify the causal question without conflating with the modeling approach (Vandenbroucke et al., 2016); hence, it allows for a simple and transparent analysis. In addition, this approach minimizes bias from potential misspecification of the outcome model (Rubin, 2004b). Furthermore, matched, stratified, and weighted analyses do not make strong assumptions of linearity in the relationship of propensity score with the outcome. If a non-parametric pre-processing of the data using propensity score methods does not reduce model dependence, it is reasonable to accept that the data do not have enough information to reliably support the causal inference by any other statistical method. In fact, this knowledge in itself should still be useful and the conclusion may be correct (Rubin, 2004b; Ho et al., 2007; Rubin, 2007; Ali et al., 2016).

### Dimension Reduction

Propensity score typically summarizes a large number of measured pretreatment covariates to a single score; hence, it is called a “summary score.” This is particularly useful in high-dimensional data with a substantially large number of pretreatment covariates compared to the number of outcome events including rare events, typical of most medication safety studies in pharmacoepidemiology (Glynn et al., 2006). In this setting, maximum likelihood estimations used in conventional regression techniques such as logistic and Cox regression require several outcome events for each parameter included in the regression model; the rule of thumb is that ≥ 10 outcome events are required per confounder included in a model (Peduzzi et al., 1995; Peduzzi et al., 1996). On the other hand, Cepeda et al. (2003) suggested using propensity score when there are fewer than eight outcomes per included covariate to effectively improve estimation.

### Doubly Robust Estimations

Generally, doubly roubst estimations (DR) estimation methods apply different procedures or models simultaneously and produce a consistent estimate of the parameter if either of the two models, not necessarily both, has been correctly specified (Imai and Ratkovic, 2014). Several applications of propensity scores have been described as DR in terms of estimating the effect of a certain treatment, including:

1. The combined use of propensity score methods (matching, regression, or weighting) with regression adjustments. These approaches use non-parametric pre-processing of the data to minimize imbalances in measured covariates and, if there are still residual differences, the covariates can be adjusted in the outcome model (Rubin and Thomas, 2000; Nguyen et al., 2017).
2. The combined use of propensity and prognostic score methods (Leacy and Stuart, 2014; Ali et al., 2018b); a prognostic score is any function of a set of covariates that when conditioned on creates independence between the potential outcome under the control (no treatment) condition and the unreduced covariates (Hansen, 2008). Hence, differences in outcomes between treated and untreated individuals can be attributed to the effect of the treatment under study. The two approaches could be combined in several ways such as full matching on a Mahalanobis distance combining the estimated propensity and prognostic scores; full matching on the estimated prognostic score within propensity score calipers; and subclassification on an estimated propensity and prognostic score grid with five subclasses, among others (Leacy and Stuart, 2014; Ali et al., 2018b). Methods combining propensity and prognostic scores were no less robust to model misspecification than single-score methods even when both prognostic and propensity score models were incorrectly specified in simulation and empirical studies (Leacy and Stuart, 2014).
3. The use of covariate balancing propensity score (CBPS) introduced by Imai and Ratkovic (2014) involves estimation of the propensity score such that the resulting covariate balance is optimized. This approach utilizes the dual characteristics of the propensity score as a covariate balancing score and the conditional probability of treatment assignment. Specifically, “the covariate balancing property (i.e., mean independence between the treatment status and measured covariates after inverse propensity score weighting) is used as condition to imply estimation of the propensity score while also incorporating the standard estimation procedure” (Imai and Ratkovic, 2014). Unlike other covariate balancing methods, a single model determines the treatment assignment mechanism and the covariate balancing weights. Once CBPS is estimated, various propensity score methods such as matching and weighting can be implemented without modification (Imai and Ratkovic, 2014). The basic idea of CBPS is optimizing covariate balance so that even when the propensity score model is misspecified, there will still be a reasonable balance of the covariates between the treatment and comparator groups. Unlike standard DR estimators, however, the CBPS approach does not require estimation of the outcome model.
4. Calculation of DR estimators using different approaches, for example, using the propensity score, predicted, and observed outcome (Ŷ and Y, respectively). This approach involves specifying regression models for the treatment (Z) and the outcome (Y) as a function of covariates (X) and combining these subject-specific values to calculate the DR estimate for each individual. First, treatment is modelled as a function of covariates to estimate propensity scores for each individual using the observed data. Second, the relationships between measured confounders and the outcome are modelled within treated and untreated groups separately. The resulting parameter estimates are then used to calculate predicted outcomes (Ŷ_1_, Ŷ_0_) for each individual in the population that is treated (setting Z = 1) and not treated (setting Z = 0) given covariate values. Third, the DR estimates of the outcome are calculated for each individual both in the presence and absence of treatment (DR_1_ and DR_0_), respectively) using the subject-specific predicted (Ŷ) and observed (Y) outcomes weighted by the propensity score. Finally, the means of DR_1_ and DR_0_ are calculated across the entire study population and these means will be used to calculate the effect of the treatment (Funk et al., 2011).

### Unmeasured Confounding

Propensity score methods, like other conventional regression methods, can account for only measured confounding factors and not unmeasured factors (Rosenbaum and Rubin, 1983). Therefore, propensity score analyses are only as good as the completeness and quality of the potential confounding variables that are available to the researcher. The only way to convince a critical reader that the study is not subject to unmeasured confounding is to have a rich set of covariates for constructing the propensity score model. Therefore, it is important to provide a detailed account of the variables collected and included in the propensity score model (Ali et al., 2015).

Modifications of the standard propensity score applications have been suggested to further reduce the risk of unmeasured confounding including the use of high-dimensional propensity score and propensity score calibration. High-dimensional propensity score refers to the use of a large number (in the range of several hundreds) of covariates to improve control of confounding; the underlying assumption is that the variables may collectively be proxies for unobserved confounding factors (Schneeweiss et al., 2009; Rassen et al., 2011). Propensity score calibration refers to the use of a “gold standard” propensity score estimated in a separate validation study, with more detailed covariate information unmeasured in the main study, to correct the main-study effect of the drug on the outcome (Stürmer et al., 2005; Stürmer et al., 2007).

Furthermore, sensitivity analyses (Rosenbaum and Rubin, 1983; Rosenbaum, 2005) are useful to assess the plausibility of the assumptions underlying the propensity score methods and how violations of them might affect the conclusions drawn (Stuart, 2010). Methods to deal with unmeasured confounding are summarized in **Figure 1**.

### Effect Modification

In estimating treatment effects, there is often an interest to explore if the effect of treatment varies among different subgroups (for example, men versus women) of the population under study, often called “treatment effect modification.” There are many ways to utilize propensity score methods to adjust for confounding in a subgroup analysis; however, common implementation of propensity score matching in the medical literature is sub-optimal (Wang et al., 2017; Ali et al., 2018a). The use of propensity score matched (PSM) cohort for subgroup analysis breaks the matched sets and might result in imbalance of covariates (Ali et al., 2018a). Depending on the frequency of treatment or outcome, small changes in the matched cohort might lead to large fluctuations for measures of association (Rassen et al., 2012).

**Figure 4:**
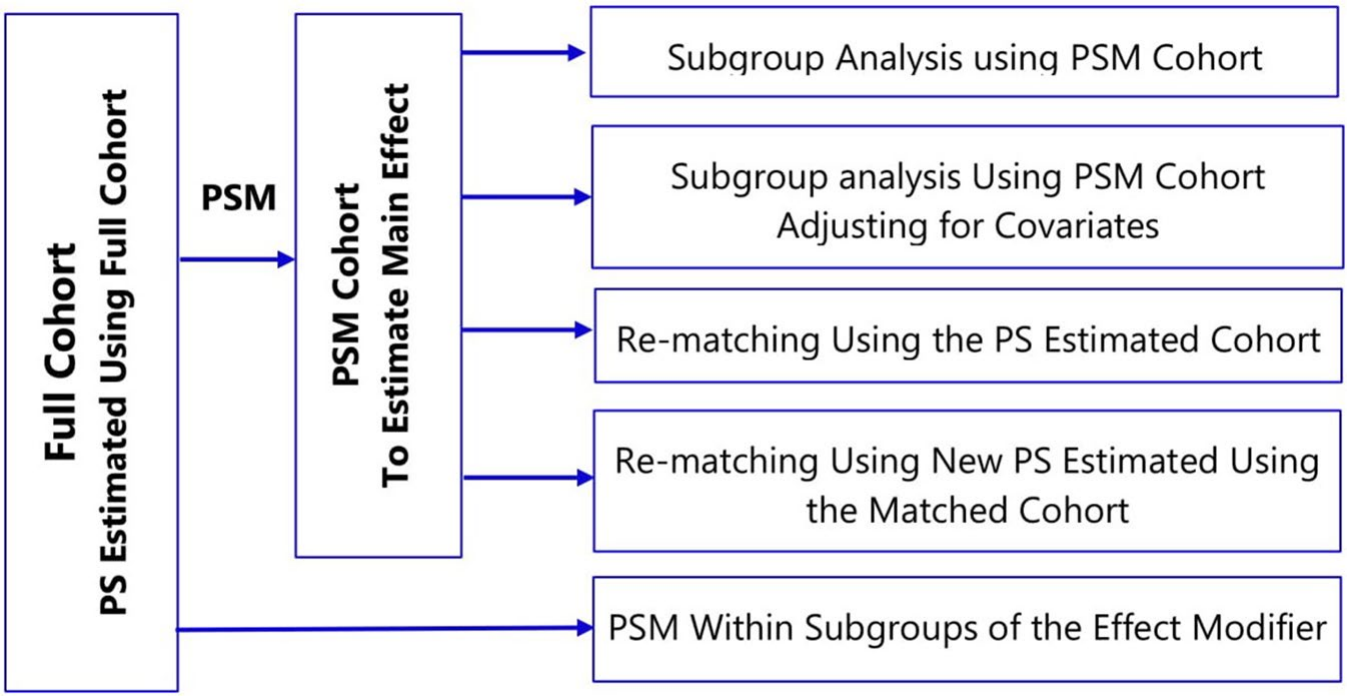
Methods to assess treatment effect modification in propensity score matching.

To account for covariate imbalances, subgroup analyses of propensity score matched cohorts involve: i) adjusting for covariates in the outcome model or ii) re-matching within the subgroups either using the propensity score estimated in the full cohort or fitting new propensity score within subgroups (**Figure 4**) (Rassen et al., 2012; Wang et al., 2017). The choice of a specific method should take into account several factors: prevalence of the treatment and the outcome; strength of association between pretreatment covariates and the treatment; the true effect size within subgroups; and the amount of confounding within the subgroups (Wang et al., 2018).

## Reporting

The credibility of any research depends on a critical assessment by others of the strengths and weaknesses in study design, conduct, and analysis. Hence, transparent and adequate reporting of critical aspects of propensity score-based analysis (Ali et al., 2015), like other observational studies, helps readers follow “what was planned, what was done, what was found, and what conclusions were drawn” (Von Elm et al., 2007). It also makes it easier for other researchers to replicate the study findings using other data sources and to judge whether and how results can be included in systematic reviews (Von Elm et al., 2007). Despite substantial methodological developments and common applications of the propensity score methods, in general, reporting on important features of the propensity score analysis is poor, incomplete, and inconsistent in the medical literature (Austin, 2008a; Ali et al., 2015; Ali et al., 2016; Wang et al., 2017). This could in part be due to a lack of standards for the conduct and reporting of propensity score based studies in guidelines. Therefore, critical items relevant to propensity score analyses should be incorporated in guidelines on the conduct and reporting of observational studies, such as the STROBE statement (Von Elm et al., 2007; Ali et al., 2015) and the ENCePP guide on methodological standards in pharmacoepidemiology (Blake et al., 2012; Ali et al., 2015) to improve the quality of the conduct and reporting of propensity score based studies (Ali et al., 2015; Ali et al., 2016). **Table 2** summarizes important consideration when planning, conducting, and reporting propensity score analysis and list of items that should be reported are summarized by Ali et al. (2016).

**Table 2:** Summary of considerations when planning, conducting, and reporting propensity score analysis.

Characteristics	What to consider	Methods available to deal with	What should or should not be done
Missing data	Missing data mechanism	Multiple imputation if missing at random (MAR)	Avoid complete case analysis and missing indicator category, the later may be biased even when MCAR assumption holds.
Variable selection	Potential confounders, intermediates, colliders	Clinical knowledge/expert opinion.	Avoid adjusting for intermediates, colliders, and strong instrumental variables the later (only when sure or suspect strong unmeasured confounding).
		Association between variables with outcome (and treatment).	Avoid the use of p-values, or step-wise variable selection methods.
		Balance diagnostics.	
Propensity score estimation	Variables included, interactions and higher order terms.	Logistic regression, Recursive partitioning, Neural network, Classification and regression trees, Random forest, and Boosting regression.	Report on the method used for estimation and variables included in the propensity score method.
Propensity score methods	The research question, the treatment effect estimand, and the extent of overlap.	Density plots of propensity scores.	Report the density plots or histograms in the propensity score distribution (preferably overlapping coefficients of the density plots).
Propensity score matching	Matching algorithm, matching with or with our replacement, and matching ratio	Exact (coarsened) matching, nearest neighbor matching (with or without caliper), stratified matching, and full matching. Matching ratio can be: 1-to-1 matching, 1-to- many matching, variable ratio matching, and full matching.	Report on the number of starting population, number matched, and number excluded (with their pre-treatment characteristics).
Propensity score stratification	Number of strata	Deciles and quintiles of propensity scores.	Report on the number of strata used and the covariate balance between treatment groups in each strata.
Regression adjustment using propensity score	Linear relationship between the outcome and the propensity core.		Report on whether linear relationship between the outcome and propensity core is checked and is fulfilled.
Inverse probability of treatment weighting	Whether there is sufficient overlap (positivity).	Weighted regression. Robust variance estimation or Bootstrapping for constructing confidence intervals.	Report on how weights are calculated, if weights are stabilized, the mean weights in both treatment groups, if trimming has been done.
Time-varying exposure	Whether there is time-varying confounding, and if any, whether it is affected by previous treatment.	Marginal Structural models using IPTW, G-formula and G-estimation of structural nested models.	If previous treatment affect time-varying confounding avoid matching, stratification and regression adjustment; apply MSM using IPTW.
Treatment effect modification	Identify potential effect modifier.	Matching on PS within strata of effect modifier, among others.	Avoid the use of stratified analysis using the PSM data without adjustment for covariates.
Multilevel treatment	Whether multilevel structure exists in the data, the number of clusters/levels	Multilevel propensity score methods.	Avoid use of single-level propensity score applications. Include multilevel structure at least in propensity score estimation or outcome analysis, preferably in both.
Multiple treatments	Number of treatment groups, whether there is order in the treatment categories (such as dosage).	Multiple matching and weighting: multinomial logistic regression, ordinal logistic regression, or generalized boosted model.	
Residual Confounding	Whether there is imbalance in covariates.	Doubly robust methods, propensity score calibration (PSC), high dimensional propensity score (HDPS) method.	Report on which method was used and why?
Unmeasured confounding	Whether there is potential unmeasured confounding, or whether the data contain proxies for unmeasured confounding.	Alternative methods such as instrumental variable methods, PSC, HDPS, or consider sensitivity analysis.	Report on the method used and the sensitivity analysis conducted.

## Conclusion

Propensity score methods will remain important design and analytic tools to estimate effects of treatment from observational data. Preferably, they should be utilized in the design stage as tools for preprocessing of the data and they should be considered complementary tools, and not replacements, to conventional regression adjustments. In fact, when appropriate, propensity score methods should be used in combination with other model-based regression techniques. In addition, propensity score methods should not be regarded as magical remedies for the inadequacies of observational studies such as residual or unmeasured confounding (Rubin and Thomas, 2000; Ali et al., 2016). The ability of propensity score methods to overcome confounding is entirely dependent on the extent to which measured variables capture potential confounding. Taking full advantage of these methods requires explicit definition of the research question and appropriate choice of the propensity score method, transparent and detailed description of all subsequent statistical analyses to be conducted, and adequate reporting of the important aspects of the propensity score analyses (Ali et al., 2016).

## Author Contributions

MA, DP-A, RF, MB, and LS contributed to the conception and design of the study. MA wrote the first draft of the manuscript. DR and NB wrote sections of the manuscript. All authors contributed to manuscript revision and read and approved the submitted version.

## Funding

This work is part of The 100 Million Brazilian Cohort project funded by the Wellcome Trust. Grant code: 202912/B/16/Z.

## Conflict of Interest Statement

LS holds research grants from GSK, Wellcome, MRC, NIHR, BHF, and Diabetes UK and is a Trustee of the British Heart Foundation. DP-A holds research grants from NIHR, AMGEN, and UCB.

The remaining authors declare that the research was conducted in the absence of any commercial or financial relationships that could be construed as a potential conflict of interest.
